# Expression and Functional Roles of Eukaryotic Initiation Factor 4A Family Proteins in Human Cancers

**DOI:** 10.3389/fcell.2021.711965

**Published:** 2021-11-19

**Authors:** Chen Xue, Xinyu Gu, Ganglei Li, Zhengyi Bao, Lanjuan Li

**Affiliations:** ^1^ State Key Laboratory for Diagnosis and Treatment of Infectious Diseases, National Clinical Research Center for Infectious Diseases, Collaborative Innovation Center for Diagnosis and Treatment of Infectious Diseases, The First Affiliated Hospital, College of Medicine, Zhejiang University, Hangzhou, China; ^2^ Department of Neurosurgery, The First Affiliated Hospital, College of Medicine, Zhejiang University, Hangzhou, China

**Keywords:** eIF4A family, human cancer, clinicopathological features, biomarkers, immune infiltrations

## Abstract

The dysregulation of mRNA translation is common in malignancies and may lead to tumorigenesis and progression. Eukaryotic initiation factor 4A (eIF4A) proteins are essential for translation, exhibit bidirectional RNA helicase function, and act as RNA-dependent ATPases. In this review, we explored the predicted structures of the three eIF4A isoforms (eIF4A1, eIF4A2, and eIF4A3), and discussed possible explanations for which function during different translation stages (initiation, mRNA localization, export, and mRNA splicing). These proteins also frequently served as targets of microRNAs (miRNAs) or long noncoding RNAs (lncRNAs) to mediate epithelial-mesenchymal transition (EMT), which was associated with tumor cell invasion and metastasis. To define the differential expression of eIF4A family members, we applied the Tumor Immune Estimation Resource website. We figured out that the eIF4A family genes were differently expressed in specific cancer types. We also found that the level of the eIF4A family genes were associated with abundant immune cells infiltration and tumor purity. The associations between eIF4A proteins and cancer patient clinicopathological features suggested that eIF4A proteins might serve as biomarkers for early tumor diagnosis, histological classification, and clinical grading/staging, providing new tools for precise and individualized cancer treatment.

## Background

Cancer is one of the most common diseases affecting human health and has imposed a heavy economic burden on society worldwide (Bray et al., 2018). As a frequent characteristic of malignancy, the dysregulation of messenger RNA (mRNA) translation may lead to tumorigenesis and progression (Bhat et al., 2015; Vadivel Gnanasundram and Fåhraeus, 2018). The translation of mRNA is a complex process that includes the steps of initiation, elongation, and termination (Dever and Green, 2012). The initiation phase is the rate-limiting step (Sonenberg and Hinnebusch, 2009; Jackson et al., 2010). The majority of evidence has confirmed that various eukaryotic initiation factors are closely associated with the genesis and prognosis of many types of human cancers (Hsieh et al., 2010; Bhat et al., 2015; Pelletier et al., 2015).

**FIGURE 1:**
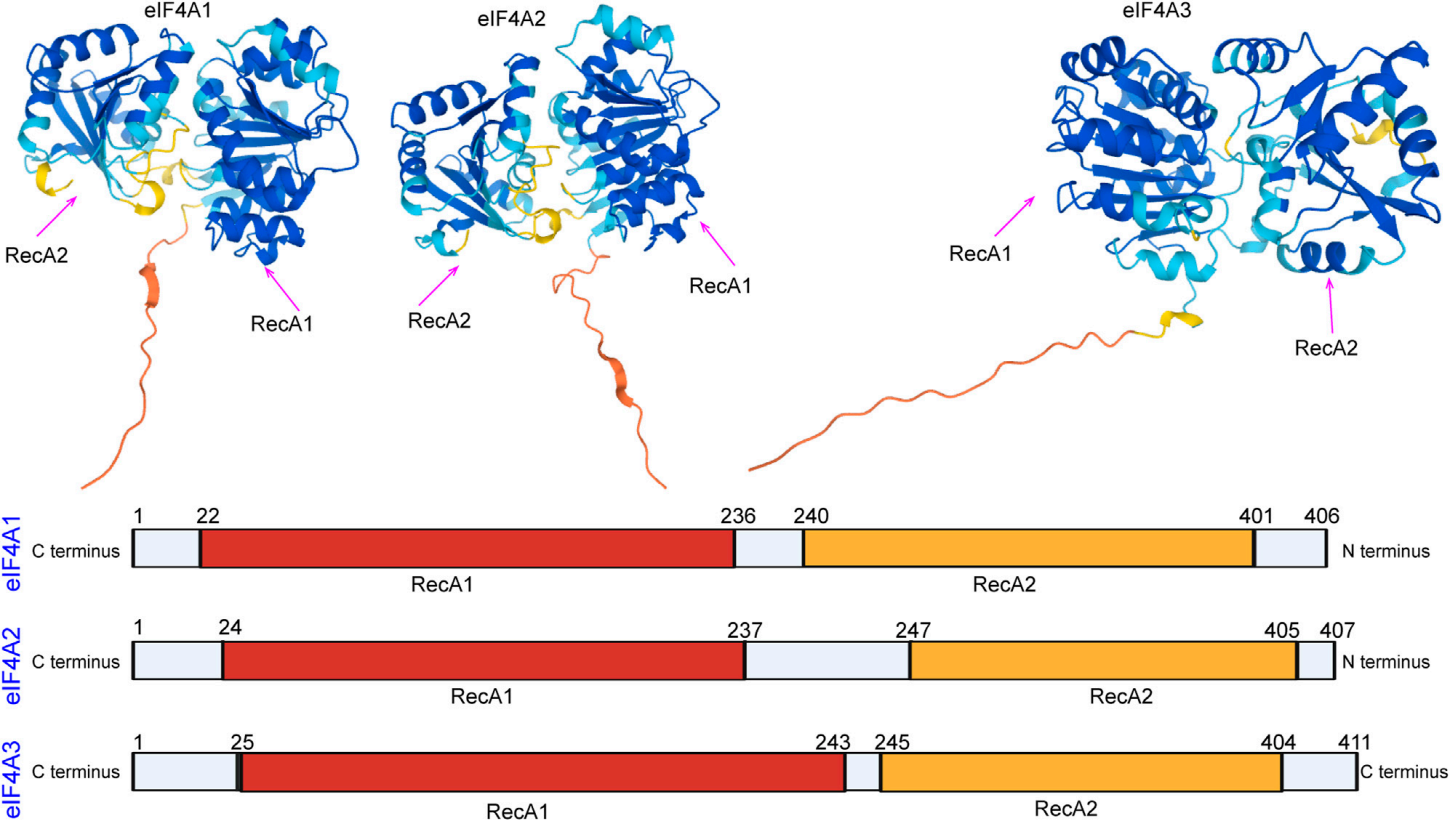
Domain organization of eIF4A1, eIF4A2, and eIF4A3. The N-terminal RecA-like domain is depicted in yellow, and the C-terminus is depicted in red. The domain organization of the eIF4A family was downloaded from the alphafold protein structure database (https://alphafold.ebi.ac.uk/).

In eukaryotes, members of the eukaryotic initiation factor 4A (eIF4A) family are essential factors for translation (Linder, 2003; Linder, 2006), and they also serve as prototypes of DEAD-box family members (Parsyan et al., 2011; Rogers et al., 2002; Rogers et al., 2001). The different eIF4A isoforms have been named as follows: eIF4A1 (DDX2A), eIF4A2 (DDX2B), and eIF4A3 (DDX48) (Iwatani-Yoshihara et al., 2017). eIF4A domains are the first determined DEAD-box protein structures that exhibit RecA-like folds (the nucleotide-binding site) and interactions between conserved motifs within the domains (Figure 1). Generally, eIF4A1 is more abundant in the cytoplasm than eIF4A2, while eIF4A3 is mainly localized in the nucleus (Lu et al., 2014). Both eIF4A1 and eIF4A2 participate in the initiation of translation. The eIF4A3 protein functions in RNA metabolism, including mRNA localization, export, and the coupling of mRNA splicing to translation (Mazloomian et al., 2019).

The ATP-dependent RNA helicase, eIF4A, plays important roles in human cancers (Wolf and Hatzfeld, 2010; Fukao et al., 2014; Sridharan et al., 2019). Alterations in the expression levels of eIF4A1, eIF4A2, and eIF4A3 have been observed in different types of malignancies and are closely associated with the clinicopathological characteristics of tumors (Ji et al., 2003) (Lin et al., 2018). With advances in the understanding of the regulation of the eIF4A family, several studies have suggested that eIF4A biomarkers could be used for human cancer diagnostics and therapies (Wang et al., 2002; Shaoyan et al., 2013a). Herein, we summarize the regulatory mechanisms and biological functions of eIF4A proteins during the process of mRNA translation. Additionally, we discuss the roles of each eIF4A isoform in tumorigenesis and cancer progression, and we propose their use as biomarkers for cancer prognosis, diagnostics, and treatment.

## Regulatory Mechanisms of the eIF4A Family

### Role of eIF4A1 in Translation Initiation

**FIGURE 2:**
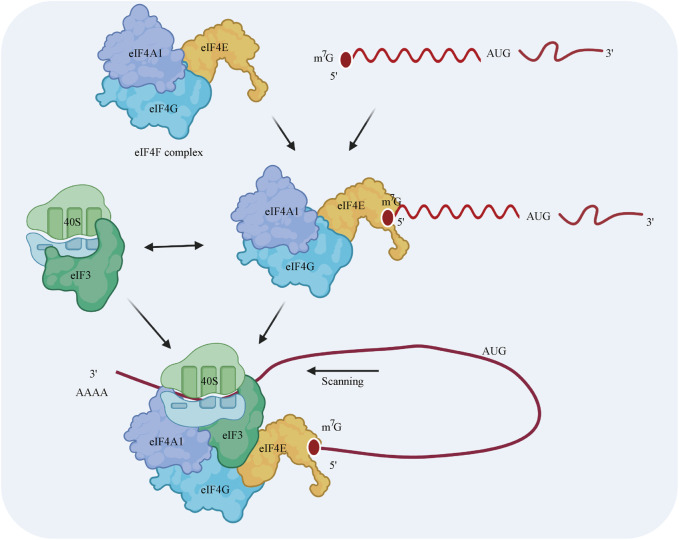
Model of the regulatory mechanism by which eIF4A1 initiates cap-dependent translation. The eIF4F cap-binding complex is composed of the eIF4A1 translation initiation factor, the eIF4G scaffolding protein, and the eIF4E m^7^G cap-binding protein. The 40S ribosomal subunit is recruited by interacting with eIF4F and eIF3 prior to the binding of eIF4F to the 5′ cap of the mRNA. This 43S preinitiation complex scans the 5′-UTR for the AUG initiation codon. During this process, eIF4A1 unwinds the stable secondary structures in the 5′-UTR of the mRNA.

eIF4A1 is a necessary component of eIF4F, which is a protein complex consisting of eIF4A1, eIF4E, and eIF4G (Jackson et al., 2010) (Merrick, 2015) (Topisirovic et al., 2011). Translational control usually occurs at the translation initiation step, in which ribosomes are recruited to the 5′ cap of the mRNA. First, eIF4E, as part of the eIF4F complex, promotes the recruitment of the 40S ribosomal subunit by interacting with the 5′ terminus of the mRNA (Siddiqui and Sonenberg, 2015). eIF4G plays a scaffolding role by interacting with both eIF4E and eIF4A1 (Lamphear et al., 1995; Mader et al., 1995). The recruitment of the 40S ribosomal subunit is induced by the interactions among eIF3, eIF4G, and the 40S subunit in mammals (except in yeast) (Jivotovskaya et al., 2006). Then, the 40S complex scans the 5′-untranslated region (UTR) for the AUG initiation codon. The elongation-competent 60S subunit is then recruited, and an elongation-competent 80S ribosome is formed. Notably, ribosomes have a weak capacity to unwind mRNA secondary structures (Takyar et al., 2005), while eIF4A1 has the ability to unwind stable secondary structures in the 5′-UTR during scanning (Sonenberg, 1988; Svitkin et al., 2001; Pestova and Kolupaeva, 2002) (Figure 2).

Prior studies have shown that the dysregulation of translation is an essential step in tumorigenesis and progression for the direct control of the selective translation and protein synthesis of oncogenic mRNA (Silvera et al., 2010; Waldron et al., 2019). The eIF4F translation initiation complex controls the translation initiation rates of many pro-oncogenic mRNAs and serves as a critical node under the regulation of the PI3K/Akt/mTOR signaling pathway (Lin et al., 2008), the mitogen-activated protein kinase signal transduction pathway, and the caspase-dependent apoptotic pathway (Blagden and Willis, 2011). As an important component of eIF4F, eIF4A1 plays a vital role in malignant transformation and progression, and recent evidence has shown that eIF4A1 is dysregulated in gastric cancer (GC) (Gao et al., 2020), colorectal cancer (Li W. et al., 2017), cervical cancer (Liang et al., 2014), hepatocellular carcinoma (Zhang et al., 2020), ovarian cancer (Zhang et al., 2018), and other cancers.

### Differences Between eIF4A2 and eIF4A1

eIF4A2 and eIF4A1 are approximately 90% identical at the amino acid level (Schütz et al., 2010) (Figure 1). Although both proteins have indistinguishable functions during translation initiation, eIF4A1 is essential for initiation, whereas eIF4A2 is not essential for initiation (Galicia-Vázquez et al., 2015). Inhibition of eIF4A1 leads to increased eIF4A2 transcription. However, eIF4A2 does not rescue the translation or cell proliferation inhibition caused by eIF4A1 inhibition (Galicia-Vázquez et al., 2012). Recent studies have found that mutations in eIF4A1 result in the repression of translation, whereas the expression of eIF4A2 mutants does not repress translation (Wilczynska et al., 2019). The amount of free functional eIF4A1 is regulated by programmed cell death 4 (PDCD4), and the abundance of eIF4A1 itself is regulated by mTOR and the carcinogen, miR-21. However, it is not clear whether inhibition of PDCD4 also affects eIF4A2 (Dorrello et al., 2006; Asangani et al., 2008).

### Regulatory Molecule of eIF4A3

**FIGURE 3:**
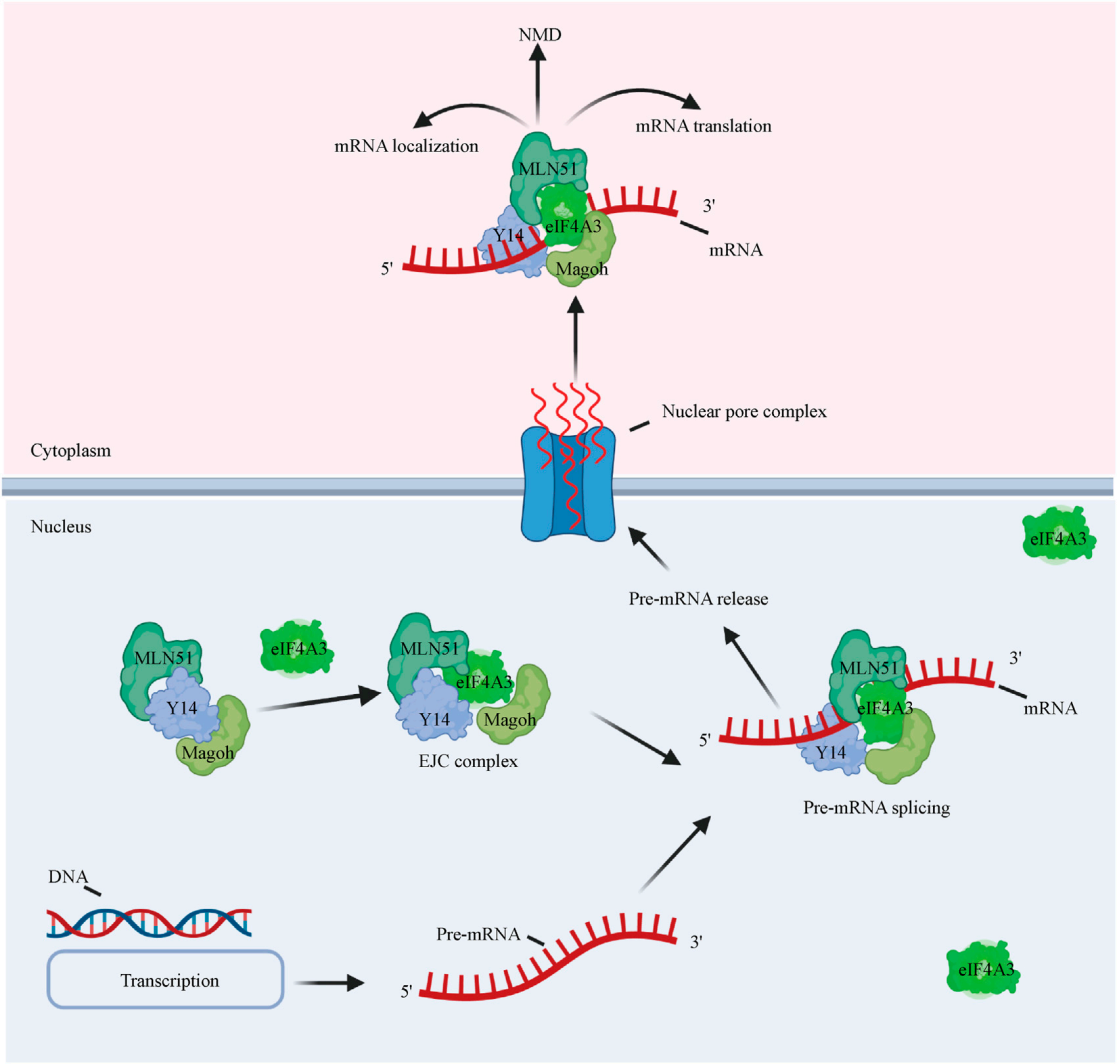
eIF4A3 affects premRNA splicing and mRNA metabolism. eIF4A3 is an essential component of the EJC and serves as a nucleation center to recruit other EJC components (i.e., the Y14/Magoh heterodimer and MLN51). The complex binds the mRNA to facilitate its translocation to the cytoplasm from the nucleus to facilitate downstream processes, such as the NMD pathway, mRNA localization, and translation.

eIF4A3 exhibits 65% amino acid identity with human eIF4A1 (Figure 1) and functions differently from eIF4A1 and eIF4A2 (Li et al., 1999). eIF4A3 has the same ATPase activity, but eIF4A3 on its own does not show helicase activity and is not involved in the initiation of translation (Noble and Song, 2007; Rozovsky et al., 2008). eIF4A3 is well known to be a component of the exon junction complex (EJC) (Le Hir and Séraphin, 2008) and serves as a nucleation center to recruit other EJC components (i.e., MLN51 and Magoh/Y14) (Andersen et al., 2006; Ballut et al., 2005; Bono et al., 2006). The EJC is a group of proteins that deposits on and accompanies mRNAs from the nucleus to the cytoplasm and coordinates premRNA splicing with downstream processes, such as nonsense-mediated decay (NMD), mRNA localization, and translation (Figure 3 **)** (Andreou and Klostermeier, 2013) (Blazquez et al., 2018). Although the mechanism by which the EJC is positioned on the mRNA is not clear, it is well established that the EJC stably binds the mRNA during premRNA splicing (Reed and Hurt, 2002; Ferraiuolo et al., 2004; Shibuya et al., 2004).

## eIF4A Expression Patterns in Cancer

The dysregulation and aberrant expression of eIF4A isoforms have been found in various tumor tissues (Raza et al., 2015; Lin et al., 2018; Wang et al., 2018). Although the exact roles of these members in tumorigenesis are not yet clear, they may be related to the dysfunction of the RNA helicase and lead to the expression of proteins formed by abnormal RNA translation (Polunovsky and Bitterman, 2006; Loh et al., 2009). We have summarized the data about eIF4A family members in various types of cancer in Table 1.

**TABLE 1:** Expression patterns and clinical significance of the eIF4A family in human malignancies.

Isoform	References	Cancer type	Expression in tumor	Clinical significance	Prognosis
eIF4A1	22415234	Endometrial cancer	High	Not studied	Not studied
	32147684	Gastric cancer	High	Poor tumor differentiation, late T stage, lymph node metastasis, advanced TNM stage	Poor prognosis
	25611378	Breast cancer	Not studied	High histological grade	Poor prognosis
	31807078	Oral squamous cell carcinoma	Not studied	Poor differentiation	Poor prognosis
	24844222	Cervical cancer	High	Advanced stage, squamous cell histology, lymph node metastasis, deep stromal invasion	Poor prognosis
	12970751	Non-small cell lung cancer	Not studied	Metastasis	Poor prognosis
eIF4A2	23867391	Non-small-cell lung cancer	Low	Low histopathological classification, early tumor grade	Favorable prognosis
	31088567	Colorectal cancer	High	distant metastasis, TNM stage IV	Poor prognosis
	31308851	Triple-negative breast cancer	High	Not studied	Not studied
	32934744	Esophageal squamous cell carcinoma	High	Not studied	Not studied
eIF4A3	29571014	Ovarian cancer	High	Not studied	Not studied
	31975383	Hepatocellular carcinoma	High	Not studied	Not studied
	32307743	Gastric cancer	Low	Not studied	Not studied

### Expression Patterns of eIF4A1 and eIF4A2 in Gastric Cancer

Gao et al. examined the mRNA expression levels of eIF4A1 in GC by employing the Gene Expression Omnibus (GEO) and showed that eIF4A1 mRNA is significantly upregulated in GC tissues compared to adjacent normal tissues (Gao et al., 2020). Similarly, immunohistochemical staining of the eIF4A1 protein in patients with GC showed that eIF4A1 protein levels are generally increased in tumor tissues (Gao et al., 2020). Wei et al. demonstrated that the expression levels of eIF4A1 protein were upregulated in 74 clinical GC samples (Wei et al., 2019), similar to the results obtained by other research teams (Li et al., 2020a). Additionally, the overexpression of eIF4A1 has been positively associated with advanced tumor-node-metastasis (TNM) stage, poor tumor differentiation, and a poor prognosis in patients with GC (Gao et al., 2020).

### Expression Patterns of eIF4A1 and eIF4A2 in Lung Cancer

Shaoyan et al. found that the mRNA expression of eIF4A2 was increased in 87.6% (148/170) of patients with nonsmall-cell lung carcinoma (NSCLC), and they observed elevated levels of eIF4A2 in tumor tissues (45.29%; 77/170) using immunohistochemistry (Shaoyan et al., 2013b). Contrary to these findings, eIF4A2 expression has been found to be low in tumor tissues but significantly related to three different clinicopathological features, namely, pathologic type, tumor grade, and overall survival (Shaoyan et al., 2013b). Furthermore, univariate and multivariate analyses have suggested that eIF4A2 is an independent prognostic factor in patients with NSCLC (Shaoyan et al., 2013b).

### Expression Patterns of eIF4A1 and eIF4A2 in Colorectal Cancer

In colorectal cancer, eIF4A1 is overexpressed in 86% (44/51) of primary colorectal tumors compared to adjacent normal tissues according to immunohistochemical staining (Li W. et al., 2017). Yang et al. reported that eIF4A1 is recruited by the long noncoding RNA, MAPKAPK5-AS1, to promote the translation of MAPK-activated protein kinase 5 (Yang et al., 2020). In addition, either eIF4A2 knockdown or inhibition by silvestrol significantly suppresses colorectal cancer invasion and migration as well as enhances sensitivity to oxaliplatin treatment both *in vitro* and *in vivo* (Chen et al., 2019).

### Expression Patterns of eIF4A1 and eIF4A2 in Cervical Cancer

eIF4A1 overexpression has been detected in 83.9% of cervical cancer tissues and is significantly related to advanced tumor stage, lymph node metastasis, squamous cell histology, deep stromal invasion, and poor survival in patients with cervical cancer (Liang et al., 2014).

### Expression Patterns of eIF4A1 and eIF4A2 in Breast Cancer

Modelska et al. reported that eIF4A1 upregulation is associated with a higher histological grade in estrogen receptor-negative breast cancer tumors, and the combination of eIF4A1 with eIF4B and eIF4E might serve as an independent predictor of prognosis in patients with breast cancer (Modelska et al., 2015b). Liu et al. found that eIF4A2 mRNA expression levels in paclitaxel-resistant breast cancer tissues are dramatically enhanced compared to those in paclitaxel-sensitive tissues (Liu et al., 2019). Functional experiments have further suggested that eIF4A2 knockdown significantly inhibits triple-negative breast cancer cell proliferation and induces apoptosis (Liu et al., 2019).

### Expression Patterns of eIF4A1 and eIF4A2 in Various Other Cancers

Zhao et al. reported that low levels of programmed cell death 4 and high levels of eIF4A1 predict poorer differentiation and a higher postoperative recurrence rate in early oral squamous cell carcinoma than in normal tissues, suggesting the roles of these proteins as independent risk factors for this type of cancer (Jiang et al., 2019). Other studies in melanoma (Eberle et al., 1997; Eberle et al., 2002), B-cell malignancies (Thompson et al., 2021), hypopharynx cancer (Xu et al., 2013), pancreatic cancer (Ma et al., 2019), and endometrioid endometrial cancer (Lomnytska et al., 2012) have indicated tumor promoter roles for the eIF4A1 protein.

In esophageal squamous cell carcinoma, eIF4A2 has been found to be more highly expressed in neoplastic tissues than in normal tissues, and patients with high expression levels of eIF4A2 tend to have a poorer prognosis (Lyu et al., 2020). Furthermore, the univariate and multivariate analyses have suggested that eIF4A2 is an independent prognostic factor in esophageal squamous cell carcinoma (Lyu et al., 2020).

### Expression Patterns of eIF4A3 in Cancers

Zhou et al. reported that the long noncoding RNA (lncRNA), HOXC-AS1, inhibits GC cell apoptosis by binding eIF4A3 in the Wnt/β-catenin signaling pathway (Zhou et al., 2020). Another study has revealed that eIF4A3 may bind the circular RNA, PVRL3 (Sun et al., 2018). Han et al. confirmed that when eIF4A3 binds lncRNA H19, the recruitment of eIF4A3 to cell cycle gene-related mRNAs is decreased (Han et al., 2016). In epithelial ovarian cancer, eIF4A3 is highly expressed in cancer tissues compared to adjacent normal tissues. Notably, eIF4A3 has been identified as a binding protein of lncRNA CASC2, thereby affecting epithelial ovarian cancer development (Zhang et al., 2018). Zhang et al. demonstrated that eIF4A3 is overexpressed in hepatocellular carcinoma. Functionally, eIF4A3 promotes cell proliferation, migration, and epithelial-mesenchymal transition (EMT) by binding WD (Trp-Asp) repeat domain 66 and miR-2113 (Zhang et al., 2020).

## mRNA Expression Levels of the eIF4A Family Based on Public Database Analysis

**FIGURE 4:**
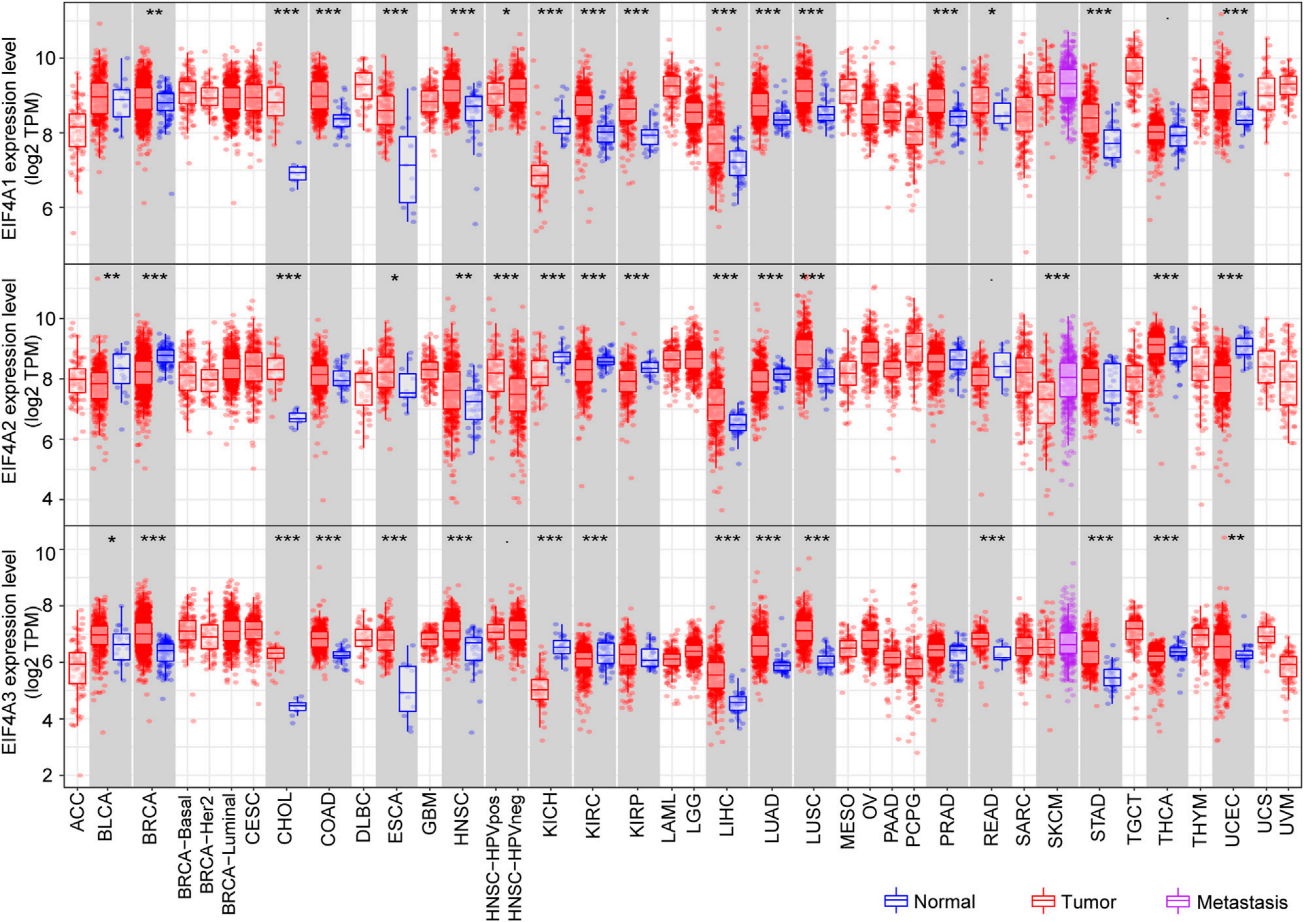
eIF4A family gene mRNA expression levels. We adopted the TIMER website to detect the expression levels of eIF4A family genes. The results showed eIF4A family gene (eIF4A1, eIF4A2, and eIF4A3) mRNA expression levels in 32 tumor tissues compared to normal tissues across multiple cancers. *p < 0.05, **p < 0.01, ***p < 0.001. Abbreviations: Kidney Renal Clear Cell Carcinoma (KIRC); Kidney Renal Papillary Cell Carcinoma (KIRP); Kidney Chromophobe (KICH); Brain Lower Grade Glioma (LGG); Glioblastoma Multiforme (GBM); Breast Invasive Carcinoma (BRCA); Lung Squamous Cell Carcinoma (LUSC); Lung Adenocarcinoma (LUAD); Rectum Adenocarcinoma (READ); Colon Adenocarcinoma (COAD); Uterine Carcinosarcoma (UCS); Uterine Corpus Endometrial Carcinoma (UCEC); Ovarian Serous Cystadenocarcinoma (OV); Head and Neck Squamous Carcinoma (HNSC); Thyroid Carcinoma (THCA); Prostate Adenocarcinoma (PRAD); Stomach Adenocarcinoma (STAD); Skin Cutaneous Melanoma (SKCM); Bladder Urothelial Carcinoma (BLCA); Liver Hepatocellular Carcinoma (LIHC); Cervical Squamous Cell Carcinoma and Endocervical Adenocarcinoma (CESC); Adrenocortical Carcinoma (ACC); Pheochromocytoma and Paraganglioma (PCPG); Sarcoma (SARC); Acute Myeloid Leukemia (LAML); Pancreatic Adenocarcinoma (PAAD); Esophageal Carcinoma (ESCA); Testicular Germ Cell Tumors (TGCT); Thymoma (THYM); Mesothelioma (MESO); Uveal Melanoma (UVM); Lymphoid Neoplasm Diffuse Large B-cell Lymphoma (DLBC); Cholangiocarcinoma (CHOL).

The Tumor Immune Estimation Resource (TIMER) (http://cistrome.dfci.harvard.edu/TIMER/) is a user-friendly website that provides comprehensive investigation of molecular characterization of tumor-immune interactions (Li et al., 2016; Li T. et al., 2017). To determine eIF4A family gene expression in normal tissues versus corresponding tumor tissues, we adopted the TIMER database and explored the eIF4A family gene mRNA expression level among multiple cancers. The eIF4A family genes associated with the RNA-seq landscape of multiple malignancies in The Cancer Genome Atlas (TCGA) are illustrated in Figure 4 **.** The results revealed that eIF4A family genes were significantly differentially expressed in various cancers compared to adjacent normal tissues (Supplementary Table S1). The patient information was similar to that in a previous study (He et al., 2021). As shown in Figure 3, eIF4A1 was significantly overexpressed in tumor tissues compared to normal control tissues, including breast invasive carcinoma (BRCA), cholangiocarcinoma (CHOL), colon adenocarcinoma (COAD), esophageal carcinoma (ESCA), head and neck squamous cell carcinoma (HNSC), kidney renal clear cell carcinoma (KIRC), kidney renal papillary cell carcinoma (KIRP), liver hepatocellular carcinoma (LIHC), lung adenocarcinoma (LUAD), lung squamous cell carcinoma (LUSC), prostate adenocarcinoma (PRAD), rectum adenocarcinoma (READ), stomach adenocarcinoma (STAD), and uterine corpus endometrial carcinoma (UCEC). eIF4A1 mRNA was downregulated in kidney chromophobe (KICH) tissues compared to normal tissues. Similarly, eIF4A2 was significantly upregulated in CHOL, COAD, ESCA, HNSC, HNSC-HPV pos, LIHC, LUAD, LUSC, and thyroid carcinoma (THCA) tissues. However, eIF4A2 was expressed at lower levels in bladder urothelial carcinoma (BLCA), BRCA, kidney chromophobe (KICH), KIRC, KIRP, skin cutaneous melanoma (SKCM), and UCEC tumor tissues compared to corresponding normal tissues. In addition, the eIF4A3 expression level in BLCA, BRCA, CHOL, COAD, ESCA, HNSC, LIHC, LUAD, LUSC, READ, STAD, THCA, and UCEC tumor tissues was increased compared to that in adjacent normal tissues. Furthermore, the eIF4A3 mRNA expression level was lower in KICH- and KIRC-related tumor tissues than in adjacent normal tissues. These studies indicated that eIF4A family genes are differentially expressed in various tumors and may function as tumor indicators in some specific types of cancers.

## eIF4A Family Genes Have a Close Relationship With Immune Cell Infiltration Across Cancers

**FIGURE 5:**
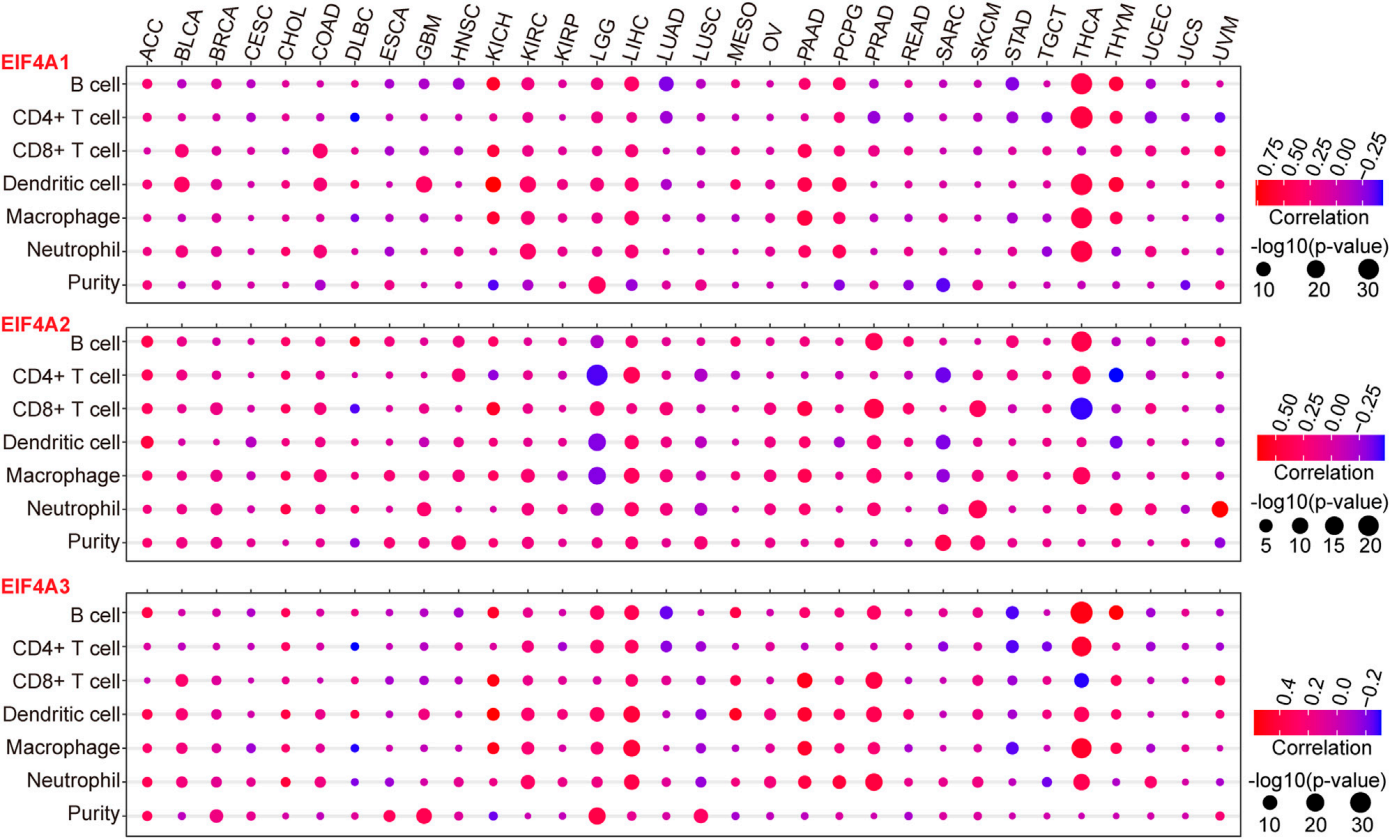
Correlation of eIF4A family gene expression with immune infiltration level and tumor purity. We adopted the TIMER website to detect the association of eIF4A family gene expression and immune infiltration levels. The mRNA expression levels of eIF4A family genes (eIF4A1, eIF4A2, and eIF4A3) were significantly correlated with the infiltration levels of various immune cells. The color of the bubble of the graph indicates the difference in each of the various types of cancers, and the bubble size indicates the statistical significance of the correlation. A correlation >0 indicates a positive association of eIF4A family genes and immune cells in various cancers. A two-tailed *p* < 0.05 was considered significant. Abbreviations: Kidney Renal Clear Cell Carcinoma (KIRC); Kidney Renal Papillary Cell Carcinoma (KIRP); Kidney Chromophobe (KICH); Brain Lower Grade Glioma (LGG); Glioblastoma Multiforme (GBM); Breast Invasive Carcinoma (BRCA); Lung Squamous Cell Carcinoma (LUSC); Lung Adenocarcinoma (LUAD); Rectum Adenocarcinoma (READ); Colon Adenocarcinoma (COAD); Uterine Carcinosarcoma (UCS); Uterine Corpus Endometrial Carcinoma (UCEC); Ovarian Serous Cystadenocarcinoma (OV); Head and Neck Squamous Carcinoma (HNSC); Thyroid Carcinoma (THCA); Prostate Adenocarcinoma (PRAD); Stomach Adenocarcinoma (STAD); Skin Cutaneous Melanoma (SKCM); Bladder Urothelial Carcinoma (BLCA); Liver Hepatocellular Carcinoma (LIHC); Cervical Squamous Cell Carcinoma and Endocervical Adenocarcinoma (CESC); Adrenocortical Carcinoma (ACC); Pheochromocytoma and Paraganglioma (PCPG); Sarcoma (SARC); Acute Myeloid Leukemia (LAML); Pancreatic Adenocarcinoma (PAAD); Esophageal Carcinoma (ESCA); Testicular Germ Cell Tumors (TGCT); Thymoma (THYM); Mesothelioma (MESO); Uveal Melanoma (UVM); Lymphoid Neoplasm Diffuse Large B-cell Lymphoma (DLBC); Cholangiocarcinoma (CHOL).

To further investigate the interactions between eIF4A family genes and the immune cell infiltration landscape and tumor purity (Gong et al., 2020; Zhang et al., 2017) in various cancer types, we employed TIMER and investigated the correlations between eIF4A family gene transcription levels and tumor infiltrating immune cells (Li et al., 2016; Li T. et al., 2017), such as B cells, CD8^+^ T cells, CD4^+^ T cells macrophages, neutrophils, and dendritic cells, as well as the tumor purity among 32 types of cancers (Supplementary Table S2), using methods described in our previous study (Xue et al., 2021). The results demonstrated that eIF4A family genes were closely correlated with immune cell infiltration in cancers. Notably, eIF4A1 had significant positive correlations with the infiltration levels of B cells, CD4^+^ T cells, macrophages, neutrophils, and dendritic cells in THCA. eIF4A1 also showed significant positive correlations with dendritic cells, CD8^+^ T cells, and neutrophils in BLCA, KICH, KIRC, LIHC, pancreatic adenocarcinoma (PAAD), pheochromocytoma and paraganglioma (PCPG), and thymoma (THYM). Tumor immune cell infiltration, which was positively correlated with the eIF4A2 mRNA expression level, was higher in KICH, THYM, COAD, PAAD, PCPG, LIHC, KICH BLCA, KIRC, and LGG than in other cancers. Dendritic cells, CD8^+^ T cells, and neutrophils had higher infiltration levels in cancers with higher eIF4A2 mRNA expression. eIF4A2 was negatively correlated with the infiltration of CD4^+^ T cells, macrophages, and dendritic cells in LGG and significantly positively correlated with the infiltration of B cells, CD4^+^ T cells, macrophages, and dendritic cells in LIHC and PRAD. In general, THCA, LGG, LIHC, PRAD, and SKCM, which exhibited significant upregulation of eIF4A2, had higher immune cell infiltration. In addition, the eIF4A3 expression level was significantly positively correlated with the infiltration of B cells, CD4^+^ T cells, macrophages, dendrites, and neutrophils in LGG, LIHC, and THCA. In THCA, eIF4A3 mRNA expression was associated with significantly higher B cell, CD4^+^ T cell, CD8^+^ T cell macrophage, and neutrophil infiltration levels. In PRAD, LIHC, LGG, and PAAD, eIF4A3 mRNA expression was associated with higher infiltration levels of B cells, CD8^+^ T cells, dendritic cells, and neutrophils (Figure 5). Our studies strongly indicated that eIF4A family genes may play specific roles in immune infiltration and tumor purity, suggesting that they may function as valuable immune evaluation indicators.

## Biological Functions of eIF4A Protein in Cancer

Most studies have demonstrated that eIF4A proteins possess protumor functions (Oblinger et al., 2016). Genome-wide studies of the eIF4A-associated translatome have revealed that eIF4A-dependent mRNAs include those that promote cell proliferation, cell survival, cell cycle progression, and angiogenesis (Rubio et al., 2014; Wolfe et al., 2014). Most studies have reported that high expression levels of eIF4A significantly promote a cancer cell malignant phenotype (proliferation, invasion, migration, and EMT) and inhibit apoptosis (Modelska et al., 2015a; Li W. et al., 2017; Liang et al., 2017; Li et al., 2020b; Gao et al., 2020).

eIF4A1 expression is regulated by circ-008035 via miR-599 binding, which ameliorates the effects of circ-008035 knockdown on GC cell proliferation and suppresses apoptosis (Li et al., 2020a). Li et al. reported that eIF4A1 is the direct target of miR-133a, which promotes colon cancer cell progression by inhibiting eIF4A1 expression (Wang et al., 2017). Similarly, the silencing of eIF4A1 in WM858 cells significantly decreases melanoma proliferation and invasion (Joyce et al., 2017). eIF4A1 has also been shown to promote the tumor cell malignant phenotype in breast (Modelska et al., 2015b), oral squamous cell (Zhao et al., 2019), and cervical (Liang et al., 2017) cancers.

Chen et al. reported that eIF4A2 dysfunction, induced by genetic knockdown or inhibition, suppresses colorectal cancer cell invasion, cell migration, and sphere formation as well as increases tissue sensitivity to oxaliplatin both *in vivo* and *in vitro* (Chen et al., 2019). In triple-negative breast cancer, miR-5195-3p upregulation increases the sensitivity of cancer cells to paclitaxel; the silencing of eIF4A2 mimics this effect, and the restoration of eIF4A2 blocks this effect (Long et al., 2019).

Han et al. reported that eIF4A3 is the binding protein of lncRNA H19, as shown by RNA-binding protein immunoprecipitation experiments, and that it participates in colorectal cancer cell proliferation via lncRNA H19 binding (Han et al., 2016). Xu et al. found that circ_cse1l is downregulated in colorectal cancer and that downregulated circ_cse1l inhibits PCNA expression by binding to eIF4A3 to inhibit the proliferation of colorectal cells (Xu et al., 2020). In epithelial ovarian cancer cells, eIF4A3 binds CASC2 and enhances cell viability, apoptosis, migration, and invasion (Zhang et al., 2018). Knockdown of eIF4A3 increases apoptosis (Zhang et al., 2018). In hepatocellular carcinoma, loss-of-function assays have shown that the silencing of eIF4A3 inhibits cell proliferation, migration, and EMT (Zhang et al., 2020). In GC tissues, eIF4A3 is downregulated compared to adjacent normal tissues, and the silencing of eIF4A3 increases lncRNA HOXC-AS1 expression, which promotes GC cell proliferation and EMT but represses apoptosis (Zhou et al., 2020). In cervical cancer, Sui et al. reported that hsa_circ_0101119 promotes cell proliferation, migration, and invasion but suppresses apoptosis in cervical cancer via an interaction with eIF4A3 to inhibit TCEAL6 expression (Sui et al., 2021).

## Conclusion

All the Members of the eIF4A family frequently serve as targets of microRNAs (miRNAs) or lncRNAs play key roles in tumor cell proliferation, invasion, and metastasis. Given the importance of mRNA translation in the development of cancer (Gingold et al., 2014), several small molecules have been shown to possess antitumor activities by targeting or inhibiting eIF4A1 (Stoneley and Willis, 2015; Howard et al., 2019; Howard et al., 2020). Previous studies have shown that the natural marine product, elatol, inhibits eIF4A1, providing a highly promising target for cancer therapy (Peters et al., 2018). Furthermore, hippuristanol, silvestrol, pateamine A, and oxo-aglaiastatin all target eIF4A1 (Itoua Maïga et al., 2019; Naineni et al., 2020; Steinberger et al., 2020). Rocaglates have been shown to possess potent antineoplastic activity both *in vivo* and *in vitro* by enhancing mRNA binding to both eIF4A1 and eIF4A2 (Chu et al., 2019). Some selective eIF4A3 inhibitors have also been identified as ATPase activation inhibitors (Ito et al., 2017a; Ito et al., 2017b). At present, inhibitors of the eIF4A family have stalled at a preclinical stage, and clinical evaluations are still lacking.

The present review presented that the eIF4A family genes were differently expressed in specific cancer types based on TIMER website, and we discussed the association between the eIF4A family genes and abundant immune cells infiltration and tumor purity, which could provide a clue for next study in the future. In addition, our findings posited functional roles of the eukaryotic initiation factor 4A family proteins in human cancer.
