# Supplementary material for: Expression and Functional Roles of Eukaryotic Initiation Factor 4A Family Proteins in Human Cancers
Source: Front Cell Dev Biol. 2021 Nov 19;9:711965. doi: 10.3389/fcell.2021.711965 (PMC8640450; doi:10.3389/fcell.2021.711965)
Supplement: Supplementary file 1 [file Table1.docx]

**Supplementary Table 1 The expression pattern of eIF4A family gene across pancancer.**

| **Tumor** | **Normal** | **p-value of eIF4A1** | **p-value of eIF4A2** | **p-value of eIF4A3** |
| --- | --- | --- | --- | --- |
| BLCA tumor | BLCA normal | 0.306681296 | 0.002151124 | 0.024764305 |
| BRCA tumor | BRCA normal | 0.001151729 | 1.47E-25 | 8.47E-32 |
| CHOL tumor | CHOL normal | 4.51E-09 | 2.71E-08 | 2.26E-09 |
| COAD tumor | COAD normal | 1.38E-14 | 0.155582612 | 2.36E-16 |
| ESCA tumor | ESCA normal | 5.11E-05 | 0.017897991 | 4.51E-07 |
| HNSC-HPV pos tumor | HNSC-HPV neg tumor | 0.010922263 | 1.00E-09 | 0.06930531 |
| HNSC tumor | HNSC normal | 7.97E-10 | 0.001378078 | 5.75E-11 |
| KICH tumor | KICH normal | 5.48E-11 | 1.65E-05 | 1.62E-12 |
| KIRC tumor | KIRC normal | 6.70E-23 | 4.11E-08 | 0.000799959 |
| KIRP tumor | KIRP normal | 1.65E-11 | 1.10E-06 | 0.311809886 |
| LIHC tumor | LIHC normal | 2.46E-07 | 2.43E-09 | 2.15E-19 |
| LUAD tumor | LUAD normal | 2.66E-11 | 0.000733425 | 6.35E-21 |
| LUSC tumor | LUSC normal | 9.81E-14 | 9.15E-14 | 1.79E-26 |
| PRAD tumor | PRAD normal | 6.57E-12 | 0.231041994 | 0.283809427 |
| READ tumor | READ normal | 0.025094288 | 0.071015388 | 0.00024726 |
| SKCM tumor | SKCM metastasis | 0.725636344 | 1.17E-10 | 0.109169097 |
| STAD tumor | STAD normal | 1.93E-09 | 0.208133477 | 4.79E-14 |
| THCA tumor | THCA normal | 0.055944255 | 4.98E-06 | 1.38E-05 |
| UCEC tumor | UCEC normal | 2.68E-09 | 3.96E-17 | 0.001956686 |
