# Supplementary material for: Expression and Functional Roles of Eukaryotic Initiation Factor 4A Family Proteins in Human Cancers
Source: Front Cell Dev Biol. 2021 Nov 19;9:711965. doi: 10.3389/fcell.2021.711965 (PMC8640450; doi:10.3389/fcell.2021.711965)
Supplement: Supplementary file 2 [file Table2.docx]

**Supplementary Table 2 The association between the expression levels of eIF4A family gene and immune infiltration level and tumor purity.**

|  |  | **eIF4A1** | | **eIF4A2** | | **eIF4A3** | |
| --- | --- | --- | --- | --- | --- | --- | --- |
| **Cancer types** | **Variable** | **Partial correction** | **p-value** | **Partial correction** | **p-value** | **Partial correction** | **p-value** |
| ACC | Purity | 0.261736629 | 0.024282037 | 0.229223499 | 0.049468952 | 0.313814001 | 0.006474739 |
| ACC | B Cell | 0.311230649 | 0.007358346 | 0.383157352 | 0.000820272 | 0.383537859 | 0.000809681 |
| ACC | CD8+ T Cell | 0.0493639 | 0.678333149 | 0.321085201 | 0.005609926 | -0.004430452 | 0.970325046 |
| ACC | CD4+ T Cell | 0.22223888 | 0.058792329 | 0.341023014 | 0.003152484 | 0.06492809 | 0.58523956 |
| ACC | Macrophage | 0.130323452 | 0.271785373 | 0.271909866 | 0.01995822 | 0.261147646 | 0.025640458 |
| ACC | Neutrophil | 0.207423344 | 0.078265016 | 0.17047112 | 0.14931789 | 0.337288004 | 0.003521954 |
| ACC | Dendritic Cell | 0.2815998 | 0.01579912 | 0.445649904 | 7.78E-05 | 0.356894942 | 0.001938883 |
| BLCA | Purity | -0.05014583 | 0.336750209 | 0.154138872 | 0.002991347 | -0.055777572 | 0.28522784 |
| BLCA | B Cell | -0.107394289 | 0.04085518 | 0.111918942 | 0.033032405 | 0.029303695 | 0.577866157 |
| BLCA | CD8+ T Cell | 0.291696421 | 1.30E-08 | 0.086273679 | 0.099364854 | 0.254791694 | 7.83E-07 |
| BLCA | CD4+ T Cell | 0.055281846 | 0.292184657 | 0.14402519 | 0.005841559 | -0.050615903 | 0.334887134 |
| BLCA | Macrophage | -0.065233258 | 0.213746445 | 0.110207576 | 0.035316927 | 0.198200621 | 0.000138121 |
| BLCA | Neutrophil | 0.24765204 | 1.78E-06 | 0.125049779 | 0.017141217 | 0.20318408 | 9.68E-05 |
| BLCA | Dendritic Cell | 0.372035415 | 2.00E-13 | -0.020335141 | 0.698600907 | 0.23277923 | 6.99E-06 |
| BRCA | Purity | 0.070798662 | 0.025533005 | 0.101307976 | 0.001375043 | 0.17713044 | 1.86E-08 |
| BRCA | B Cell | 0.097851722 | 0.002198734 | -0.03004389 | 0.348197427 | 0.044196972 | 0.167472524 |
| BRCA | CD8+ T Cell | 0.073126181 | 0.022332383 | 0.12652044 | 7.39E-05 | 0.080485894 | 0.011892471 |
| BRCA | CD4+ T Cell | 0.03180491 | 0.324412011 | 0.051799962 | 0.108356722 | 0.018739797 | 0.561556475 |
| BRCA | Macrophage | 0.070840176 | 0.026350633 | 0.110063967 | 0.000546359 | 0.076053907 | 0.017082549 |
| BRCA | Neutrophil | 0.108084464 | 0.000842197 | 0.110058834 | 0.000674116 | 0.111518696 | 0.00057048 |
| BRCA | Dendritic Cell | 0.117846728 | 0.000270413 | 0.002918572 | 0.928378142 | 0.098704491 | 0.002308931 |
| CESC | Purity | 0.029338929 | 0.626202726 | 0.077437089 | 0.198006987 | 0.124347213 | 0.038266472 |
| CESC | B Cell | -0.106788849 | 0.076003511 | -0.012775166 | 0.832365821 | -0.088492127 | 0.141825777 |
| CESC | CD8+ T Cell | 0.055093021 | 0.363628463 | -0.029801015 | 0.623321412 | -0.000928403 | 0.987794803 |
| CESC | CD4+ T Cell | -0.131010646 | 0.029257759 | -0.002506211 | 0.966878867 | -0.025732064 | 0.669815761 |
| CESC | Macrophage | 0.000235018 | 0.996893217 | -0.087868092 | 0.144668053 | -0.14046953 | 0.01933982 |
| CESC | Neutrophil | 0.016850317 | 0.780093796 | -0.03628513 | 0.547592166 | 0.122801492 | 0.041119993 |
| CESC | Dendritic Cell | 0.017930154 | 0.766810071 | -0.165190208 | 0.005944739 | 0.025635252 | 0.671550035 |
| CHOL | Purity | 0.043769311 | 0.799905758 | -0.009397529 | 0.956619378 | 0.032826984 | 0.849260683 |
| CHOL | B Cell | 0.092950204 | 0.595361969 | 0.268833564 | 0.118385825 | 0.323945051 | 0.057639014 |
| CHOL | CD8+ T Cell | -0.087930723 | 0.615465313 | 0.316651917 | 0.063836981 | 0.137586535 | 0.430596834 |
| CHOL | CD4+ T Cell | 0.129599739 | 0.458078222 | 0.267381079 | 0.120464916 | 0.290546859 | 0.090413639 |
| CHOL | Macrophage | 0.098816548 | 0.572236688 | 0.333527237 | 0.050231656 | 0.253546413 | 0.141653226 |
| CHOL | Neutrophil | 0.336620054 | 0.0480104 | 0.40073274 | 0.017050752 | 0.425828479 | 0.010758108 |
| CHOL | Dendritic Cell | 0.23963475 | 0.165591266 | 0.147034265 | 0.39930106 | 0.392855291 | 0.019569848 |
| COAD | Purity | -0.162811192 | 0.000978936 | 0.07267732 | 0.143293319 | -0.040259616 | 0.417918549 |
| COAD | B Cell | 0.023370458 | 0.639535108 | 0.105612836 | 0.033824928 | 0.04923221 | 0.323599631 |
| COAD | CD8+ T Cell | 0.327397566 | 1.34E-11 | 0.185980281 | 0.00016405 | -0.001062037 | 0.982979642 |
| COAD | CD4+ T Cell | -0.060443019 | 0.22658183 | 0.073746362 | 0.139939558 | 0.045723937 | 0.360514224 |
| COAD | Macrophage | 0.061606164 | 0.216605146 | 0.204705118 | 3.39E-05 | 0.095272483 | 0.055700181 |
| COAD | Neutrophil | 0.269177265 | 4.38E-08 | 0.109815514 | 0.027889069 | 0.184640652 | 0.000200886 |
| COAD | Dendritic Cell | 0.281936204 | 8.80E-09 | 0.122427887 | 0.014038592 | 0.147500089 | 0.003032846 |
| DLBC | Purity | 0.152393315 | 0.335316981 | -0.286791248 | 0.065559546 | 0.086086011 | 0.587768596 |
| DLBC | B Cell | 0.15954962 | 0.527134004 | 0.512945184 | 0.029489762 | 0.170369696 | 0.499108266 |
| DLBC | CD8+ T Cell | 0.144565706 | 0.531829626 | -0.418429807 | 0.059059836 | 0.184442834 | 0.423496516 |
| DLBC | CD4+ T Cell | -0.442898312 | 0.044354958 | -0.003254363 | 0.988829876 | -0.306435097 | 0.176680883 |
| DLBC | Macrophage | -0.298346734 | 0.188968894 | 0.140415298 | 0.543796213 | -0.295635091 | 0.193214803 |
| DLBC | Neutrophil | 0.06249801 | 0.78783165 | 0.246989069 | 0.28042136 | -0.200013362 | 0.384691813 |
| DLBC | Dendritic Cell | 0.340533511 | 0.130907679 | 0.112298251 | 0.627930607 | 0.374412599 | 0.094489971 |
| ESCA | Purity | 0.19482243 | 0.008585306 | 0.213397865 | 0.003921796 | 0.31128126 | 2.00E-05 |
| ESCA | B Cell | -0.172302904 | 0.02108927 | 0.171977716 | 0.021337228 | -0.045271259 | 0.547338243 |
| ESCA | CD8+ T Cell | -0.163626933 | 0.028177531 | 0.016865373 | 0.822203075 | -0.107861305 | 0.149517529 |
| ESCA | CD4+ T Cell | -0.050121121 | 0.505221963 | 0.02860287 | 0.703888163 | -0.014955643 | 0.842495726 |
| ESCA | Macrophage | -0.094738949 | 0.205853891 | 0.219430535 | 0.003080223 | 0.0185464 | 0.804820144 |
| ESCA | Neutrophil | -0.176978351 | 0.017470013 | 0.016213326 | 0.82896954 | -0.141198829 | 0.058668833 |
| ESCA | Dendritic Cell | -0.006409437 | 0.931948631 | 0.024188256 | 0.747221017 | -0.045277649 | 0.546148845 |
| GBM | Purity | 0.001663592 | 0.972916081 | 0.146149984 | 0.002710487 | 0.349355612 | 1.80E-13 |
| GBM | B Cell | -0.160302064 | 0.001005932 | 0.045941427 | 0.348780788 | -0.073089525 | 0.13574111 |
| GBM | CD8+ T Cell | -0.10343117 | 0.034515645 | 0.109881486 | 0.024664854 | -0.105044822 | 0.031780647 |
| GBM | CD4+ T Cell | -0.033425185 | 0.495540263 | -0.004747883 | 0.922900668 | -0.072907563 | 0.136720314 |
| GBM | Macrophage | -0.085096813 | 0.082254561 | 0.118743394 | 0.015138692 | -0.036123857 | 0.461376095 |
| GBM | Neutrophil | 0.021855491 | 0.655921095 | 0.24363931 | 4.60E-07 | 0.037672299 | 0.442381297 |
| GBM | Dendritic Cell | 0.377420028 | 1.34E-15 | -0.11054328 | 0.023807236 | 0.186447554 | 0.00012598 |
| HNSC | Purity | 0.034220892 | 0.448378923 | 0.237908686 | 8.99E-08 | 0.082791414 | 0.066245973 |
| HNSC | B Cell | -0.186484517 | 4.24E-05 | 0.151722473 | 0.00089781 | -0.111406777 | 0.015024306 |
| HNSC | CD8+ T Cell | -0.083625138 | 0.068907118 | 0.013607255 | 0.767625404 | -0.047193576 | 0.305204971 |
| HNSC | CD4+ T Cell | -0.018920617 | 0.679250453 | 0.207347597 | 4.63E-06 | 0.056649088 | 0.215390533 |
| HNSC | Macrophage | 0.028917973 | 0.526065351 | 0.169458586 | 0.000182794 | -0.017473362 | 0.701682056 |
| HNSC | Neutrophil | 0.1043303 | 0.022392142 | 0.003334071 | 0.941981775 | 0.117765811 | 0.009889227 |
| HNSC | Dendritic Cell | -0.008273928 | 0.856225197 | 0.106946786 | 0.018843836 | 0.02185798 | 0.632157617 |
| KICH | Purity | -0.381130842 | 0.001592655 | 0.217726349 | 0.079061796 | -0.230434194 | 0.062684319 |
| KICH | B Cell | 0.627760456 | 2.18E-08 | 0.279820252 | 0.02397603 | 0.474536509 | 6.51E-05 |
| KICH | CD8+ T Cell | 0.548199275 | 2.28E-06 | 0.525202165 | 7.06E-06 | 0.528056825 | 6.16E-06 |
| KICH | CD4+ T Cell | 0.128997947 | 0.305777815 | -0.295391727 | 0.016899433 | 0.064771182 | 0.608225303 |
| KICH | Macrophage | 0.581342381 | 3.82E-07 | 0.305927737 | 0.013199355 | 0.512362589 | 1.28E-05 |
| KICH | Neutrophil | 0.117610996 | 0.350791338 | -0.013585184 | 0.914466107 | 0.15469623 | 0.218538421 |
| KICH | Dendritic Cell | 0.767203019 | 9.13E-14 | 0.174259628 | 0.165041468 | 0.578523147 | 4.48E-07 |
| KIRC | Purity | -0.161036127 | 0.000511487 | 0.16276542 | 0.000443941 | -0.003227871 | 0.944836245 |
| KIRC | B Cell | 0.239798371 | 2.00E-07 | 0.048174715 | 0.303060424 | 0.129850231 | 0.005333528 |
| KIRC | CD8+ T Cell | 0.188567287 | 7.16E-05 | 0.113893937 | 0.017098815 | 0.142320488 | 0.002833509 |
| KIRC | CD4+ T Cell | 0.156644345 | 0.000748033 | 0.091398746 | 0.050106546 | 0.202452359 | 1.21E-05 |
| KIRC | Macrophage | 0.274460335 | 3.49E-09 | 0.226065163 | 1.34E-06 | 0.236895564 | 3.93E-07 |
| KIRC | Neutrophil | 0.354290485 | 5.44E-15 | 0.179544424 | 0.000111839 | 0.283423086 | 6.57E-10 |
| KIRC | Dendritic Cell | 0.361199705 | 1.81E-15 | 0.076298116 | 0.104079994 | 0.252399124 | 4.82E-08 |
| KIRP | Purity | 0.036863328 | 0.554794968 | 0.06433665 | 0.302324621 | -0.037503375 | 0.547938764 |
| KIRP | B Cell | 0.087640538 | 0.162089708 | 0.071563194 | 0.25392362 | 0.034276415 | 0.585135228 |
| KIRP | CD8+ T Cell | 0.101625609 | 0.103388871 | 0.024998201 | 0.689419279 | 0.100132765 | 0.108582805 |
| KIRP | CD4+ T Cell | -0.008106434 | 0.896899932 | -0.080570651 | 0.197060977 | -0.119414761 | 0.055412047 |
| KIRP | Macrophage | 0.133525306 | 0.035219064 | -0.142129394 | 0.024904726 | 0.044103679 | 0.488447809 |
| KIRP | Neutrophil | 0.163902747 | 0.008345905 | 0.138384734 | 0.026236073 | 0.147925721 | 0.017425895 |
| KIRP | Dendritic Cell | 0.213362696 | 0.000588727 | 0.073258132 | 0.242821313 | 0.197341338 | 0.001507309 |
| LGG | Purity | 0.386919269 | 1.49E-18 | 0.124541659 | 0.00634807 | 0.377823578 | 1.06E-17 |
| LGG | B Cell | 0.213465191 | 2.49E-06 | -0.195702958 | 1.64E-05 | 0.276681184 | 7.56E-10 |
| LGG | CD8+ T Cell | 0.137946979 | 0.002506713 | 0.237756095 | 1.44E-07 | 0.065624924 | 0.151983727 |
| LGG | CD4+ T Cell | 0.191441064 | 2.61E-05 | -0.424957379 | 2.70E-22 | 0.259020436 | 9.78E-09 |
| LGG | Macrophage | 0.165997305 | 0.000288028 | -0.330846634 | 1.52E-13 | 0.229557572 | 4.49E-07 |
| LGG | Neutrophil | 0.135551318 | 0.00307477 | -0.18920418 | 3.32E-05 | 0.17030349 | 0.000192107 |
| LGG | Dendritic Cell | 0.26454107 | 4.60E-09 | -0.330624361 | 1.33E-13 | 0.28989334 | 1.14E-10 |
| LIHC | Purity | -0.225147949 | 2.37E-05 | 0.217608354 | 4.46E-05 | 0.11884391 | 0.027072961 |
| LIHC | B Cell | 0.352831962 | 1.60E-11 | 0.20529074 | 0.00012575 | 0.349904915 | 2.42E-11 |
| LIHC | CD8+ T Cell | 0.280204878 | 1.37E-07 | 0.142643916 | 0.008246358 | 0.25578855 | 1.64E-06 |
| LIHC | CD4+ T Cell | 0.173257843 | 0.001254498 | 0.347934353 | 3.17E-11 | 0.302309138 | 1.06E-08 |
| LIHC | Macrophage | 0.341835579 | 8.84E-11 | 0.318403525 | 1.79E-09 | 0.433976211 | 4.30E-17 |
| LIHC | Neutrophil | 0.296827254 | 1.90E-08 | 0.272962798 | 2.61E-07 | 0.374260628 | 6.51E-13 |
| LIHC | Dendritic Cell | 0.318355648 | 1.91E-09 | 0.268813171 | 4.87E-07 | 0.421369494 | 4.57E-16 |
| LUAD | Purity | 0.080898756 | 0.072422177 | 0.076744917 | 0.088393504 | 0.045354701 | 0.314404686 |
| LUAD | B Cell | -0.299767623 | 1.66E-11 | 0.068506291 | 0.132321533 | -0.234878798 | 1.72E-07 |
| LUAD | CD8+ T Cell | -0.016519833 | 0.716120202 | 0.20234223 | 6.78E-06 | 0.113560862 | 0.01215037 |
| LUAD | CD4+ T Cell | -0.221241941 | 8.84E-07 | 0.077413844 | 0.088895191 | -0.178734447 | 7.69E-05 |
| LUAD | Macrophage | -0.045347045 | 0.318958283 | 0.192012174 | 2.07E-05 | 0.002821884 | 0.950574534 |
| LUAD | Neutrophil | 0.009638204 | 0.832670835 | 0.180297718 | 6.75E-05 | 0.097243218 | 0.032624991 |
| LUAD | Dendritic Cell | -0.158096055 | 0.00045563 | 0.131597211 | 0.003587809 | -0.041372941 | 0.361766179 |
| LUSC | Purity | 0.174477133 | 0.000126029 | 0.200801386 | 9.70E-06 | 0.284588794 | 2.34E-10 |
| LUSC | B Cell | -0.115287311 | 0.012196669 | 0.052346215 | 0.256369579 | -0.008415058 | 0.855315646 |
| LUSC | CD8+ T Cell | -0.0802675 | 0.080533132 | -0.063139796 | 0.169489704 | -0.098544517 | 0.03176956 |
| LUSC | CD4+ T Cell | -0.067062074 | 0.144461551 | -0.197501214 | 1.45E-05 | -0.13861287 | 0.002464505 |
| LUSC | Macrophage | -0.073343331 | 0.109644081 | -0.097670202 | 0.032953131 | -0.130645124 | 0.004261789 |
| LUSC | Neutrophil | -0.013583865 | 0.767536052 | -0.185310655 | 4.75E-05 | -0.156913266 | 0.000590919 |
| LUSC | Dendritic Cell | -0.040863314 | 0.374713369 | -0.154403693 | 0.000743596 | -0.160842201 | 0.000439008 |
| MESO | Purity | -0.03061634 | 0.779603958 | 0.131497368 | 0.227485782 | -0.118869544 | 0.275673319 |
| MESO | B Cell | 0.188639354 | 0.085711377 | 0.236452823 | 0.030352357 | 0.380648306 | 0.000354422 |
| MESO | CD8+ T Cell | 0.166836256 | 0.129312018 | 0.029847047 | 0.787534275 | 0.346191512 | 0.001256562 |
| MESO | CD4+ T Cell | -0.054675239 | 0.621328721 | -0.163181837 | 0.138037864 | -0.036340507 | 0.742772498 |
| MESO | Macrophage | -0.117973601 | 0.285167813 | 0.173088077 | 0.115367784 | -0.066442204 | 0.548178045 |
| MESO | Neutrophil | -0.001387551 | 0.990005508 | -0.018677894 | 0.866083446 | 0.193496968 | 0.077805373 |
| MESO | Dendritic Cell | 0.32446283 | 0.002602983 | -0.027624054 | 0.803026146 | 0.48998495 | 2.24E-06 |
| OV | Purity | -0.019071285 | 0.675565903 | 0.107346188 | 0.018160014 | -0.0419998 | 0.356521647 |
| OV | B Cell | 0.022710059 | 0.619669209 | 0.058726677 | 0.199005144 | 0.028288864 | 0.536386992 |
| OV | CD8+ T Cell | 0.050254184 | 0.271838848 | 0.16743079 | 0.000228969 | 0.033429075 | 0.464969128 |
| OV | CD4+ T Cell | -0.02257325 | 0.621780463 | -0.020394584 | 0.655810857 | 0.094420701 | 0.038651111 |
| OV | Macrophage | 0.112261147 | 0.013859314 | 0.171826264 | 0.000155024 | 0.096298484 | 0.034927561 |
| OV | Neutrophil | 0.118076555 | 0.009618628 | 0.146154851 | 0.001322728 | 0.20313635 | 7.27E-06 |
| OV | Dendritic Cell | 0.112092892 | 0.014003349 | 0.136626463 | 0.002703264 | 0.192227417 | 2.23E-05 |
| PAAD | Purity | -0.02714461 | 0.723738007 | 0.065192355 | 0.39551438 | -0.056701261 | 0.460027075 |
| PAAD | B Cell | 0.317829205 | 2.27E-05 | 0.166381608 | 0.029634112 | 0.251392055 | 0.000910892 |
| PAAD | CD8+ T Cell | 0.436591763 | 2.38E-09 | 0.412722958 | 2.03E-08 | 0.510994875 | 9.26E-13 |
| PAAD | CD4+ T Cell | 0.076711165 | 0.321536468 | -0.043650504 | 0.573085114 | -0.05558622 | 0.472872598 |
| PAAD | Macrophage | 0.517436285 | 4.28E-13 | 0.366127525 | 8.45E-07 | 0.46098062 | 2.23E-10 |
| PAAD | Neutrophil | 0.363958259 | 9.91E-07 | 0.252524758 | 0.000861806 | 0.397821396 | 7.12E-08 |
| PAAD | Dendritic Cell | 0.46932095 | 9.48E-11 | 0.249364276 | 0.001005228 | 0.457255094 | 3.24E-10 |
| PCPG | Purity | -0.272315816 | 0.000355632 | 0.151662678 | 0.049708531 | 0.066025661 | 0.395142013 |
| PCPG | B Cell | 0.379463677 | 4.25E-07 | 0.076467296 | 0.326005241 | 0.182835561 | 0.018031017 |
| PCPG | CD8+ T Cell | 0.27288373 | 0.000359844 | 0.095322385 | 0.220433289 | 0.150445016 | 0.052302525 |
| PCPG | CD4+ T Cell | 0.272935897 | 0.000358872 | -0.082871387 | 0.28700119 | 0.113663825 | 0.143584525 |
| PCPG | Macrophage | 0.347093467 | 4.62E-06 | 0.071081192 | 0.362796859 | 0.21213759 | 0.006072334 |
| PCPG | Neutrophil | 0.4156002 | 2.34E-08 | 0.017743787 | 0.819960393 | 0.418192679 | 1.87E-08 |
| PCPG | Dendritic Cell | 0.468573918 | 1.70E-10 | -0.21273511 | 0.005777272 | 0.289585744 | 0.000147154 |
| PRAD | Purity | 0.083557825 | 0.088351489 | -0.029045453 | 0.554206001 | 0.004974494 | 0.919329984 |
| PRAD | B Cell | -0.112040967 | 0.022939581 | 0.34739499 | 3.95E-13 | 0.291118003 | 1.72E-09 |
| PRAD | CD8+ T Cell | 0.190772732 | 9.03E-05 | 0.410839539 | 2.26E-18 | 0.394294883 | 6.33E-17 |
| PRAD | CD4+ T Cell | -0.241287711 | 7.42E-07 | 0.047604427 | 0.335699325 | 0.027376869 | 0.57997014 |
| PRAD | Macrophage | -0.074685615 | 0.128301308 | 0.272507505 | 1.62E-08 | 0.228376304 | 2.52E-06 |
| PRAD | Neutrophil | -0.023514357 | 0.633315295 | 0.225228391 | 3.68E-06 | 0.416119839 | 9.11E-19 |
| PRAD | Dendritic Cell | 0.016432205 | 0.738558925 | 0.247234534 | 3.38E-07 | 0.350956385 | 1.79E-13 |
| READ | Purity | -0.248916602 | 0.003020538 | -0.067838218 | 0.425802894 | -0.107102903 | 0.207841351 |
| READ | B Cell | 0.092927471 | 0.276567947 | 0.20547945 | 0.015239226 | 0.071794635 | 0.400971637 |
| READ | CD8+ T Cell | 0.157673503 | 0.063772106 | 0.26240536 | 0.001804526 | -0.041588991 | 0.626889448 |
| READ | CD4+ T Cell | -0.209943951 | 0.013117571 | -0.11141223 | 0.191641519 | 0.039053569 | 0.648065587 |
| READ | Macrophage | -0.103027248 | 0.227463273 | 0.08848954 | 0.30025395 | -0.10619422 | 0.213416038 |
| READ | Neutrophil | 0.180749682 | 0.033878002 | -0.001794351 | 0.983335689 | 0.136266824 | 0.111013294 |
| READ | Dendritic Cell | 0.07796622 | 0.361612296 | 0.135562522 | 0.111569016 | 0.264247869 | 0.001670137 |
| SARC | Purity | -0.371164105 | 2.03E-09 | 0.392675276 | 1.86E-10 | 0.060266947 | 0.347547558 |
| SARC | B Cell | -0.079581879 | 0.220273608 | 0.034439391 | 0.596260374 | 0.114878037 | 0.076306104 |
| SARC | CD8+ T Cell | -0.020701291 | 0.749678833 | 0.010386702 | 0.87282253 | -0.012651667 | 0.845406013 |
| SARC | CD4+ T Cell | -0.055051288 | 0.396854112 | -0.373893404 | 2.40E-09 | -0.174060621 | 0.006987783 |
| SARC | Macrophage | 0.118384682 | 0.070063285 | -0.287893206 | 7.29E-06 | 0.008543505 | 0.896350067 |
| SARC | Neutrophil | 0.072793534 | 0.259304858 | -0.156808754 | 0.01461154 | 0.132835065 | 0.038933522 |
| SARC | Dendritic Cell | 0.05324005 | 0.410624898 | -0.340665913 | 5.82E-08 | -0.029863557 | 0.644583649 |
| SKCM | Purity | 0.148347768 | 0.001453864 | 0.254435153 | 3.36E-08 | 0.06341913 | 0.175454537 |
| SKCM | B Cell | -0.045604629 | 0.335511156 | -0.005670473 | 0.904731137 | 0.142449893 | 0.002510291 |
| SKCM | CD8+ T Cell | -0.082134758 | 0.085989598 | 0.317468306 | 1.03E-11 | 0.160647557 | 0.000739664 |
| SKCM | CD4+ T Cell | -0.094162825 | 0.046874245 | 0.112366045 | 0.017601833 | 0.074844077 | 0.114476211 |
| SKCM | Macrophage | -0.018084339 | 0.701073803 | 0.158367719 | 0.000717705 | 0.063077789 | 0.180193202 |
| SKCM | Neutrophil | -0.005274686 | 0.910956915 | 0.357439541 | 4.55E-15 | 0.173764775 | 0.000205353 |
| SKCM | Dendritic Cell | -0.006853332 | 0.885240615 | 0.077576923 | 0.10179908 | 0.156935705 | 0.000882258 |
| STAD | Purity | 0.045471152 | 0.376732371 | 0.095383993 | 0.063241789 | 0.011678271 | 0.82049438 |
| STAD | B Cell | -0.289732058 | 1.44E-08 | 0.198079383 | 0.000128098 | -0.266034329 | 2.14E-07 |
| STAD | CD8+ T Cell | 0.070085916 | 0.178553357 | -0.073707347 | 0.157092815 | -0.138415237 | 0.007668958 |
| STAD | CD4+ T Cell | -0.218254274 | 2.53E-05 | 0.145868024 | 0.005173239 | -0.261069143 | 4.07E-07 |
| STAD | Macrophage | -0.178462727 | 0.000562853 | 0.157845937 | 0.002326301 | -0.252266937 | 8.84E-07 |
| STAD | Neutrophil | 0.115587 | 0.02599296 | -0.023755383 | 0.64833151 | -0.044780829 | 0.389755656 |
| STAD | Dendritic Cell | 0.04984298 | 0.338365848 | 0.030367762 | 0.559836038 | -0.118220823 | 0.022762254 |
| TGCT | Purity | -0.036760691 | 0.657352634 | 0.104555516 | 0.205994298 | 0.014513576 | 0.86101881 |
| TGCT | B Cell | 0.022383097 | 0.787854574 | 0.041781075 | 0.615341445 | -0.008983341 | 0.914003955 |
| TGCT | CD8+ T Cell | 0.157307423 | 0.057062239 | 0.129936376 | 0.116740367 | 0.130894879 | 0.114043941 |
| TGCT | CD4+ T Cell | -0.296346407 | 0.00028132 | 0.125124047 | 0.132374356 | -0.212700203 | 0.009950655 |
| TGCT | Macrophage | -0.145591505 | 0.078488948 | 0.04554431 | 0.583852744 | -0.014787461 | 0.858905501 |
| TGCT | Neutrophil | -0.23620937 | 0.003972819 | 0.082848462 | 0.318461057 | -0.225203067 | 0.006098207 |
| TGCT | Dendritic Cell | 0.085013298 | 0.307615426 | 0.122874075 | 0.139531948 | 0.181114832 | 0.028688974 |
| THCA | Purity | -0.058587064 | 0.195889549 | 0.034254673 | 0.449787949 | -0.003665485 | 0.935562077 |
| THCA | B Cell | 0.508062308 | 5.39E-33 | 0.402713808 | 3.22E-20 | 0.530775562 | 2.21E-36 |
| THCA | CD8+ T Cell | -0.090778741 | 0.045252483 | -0.446988337 | 2.70E-25 | -0.293803198 | 3.75E-11 |
| THCA | CD4+ T Cell | 0.531706214 | 5.81E-37 | 0.343844894 | 5.46E-15 | 0.460632096 | 5.29E-27 |
| THCA | Macrophage | 0.498562907 | 5.01E-32 | 0.316960012 | 7.52E-13 | 0.472162843 | 1.83E-28 |
| THCA | Neutrophil | 0.51227505 | 5.29E-34 | 0.087750883 | 0.052715098 | 0.355037201 | 6.07E-16 |
| THCA | Dendritic Cell | 0.515495014 | 2.81E-34 | 0.096262024 | 0.034056266 | 0.309516258 | 3.15E-12 |
| THYM | Purity | -0.080821066 | 0.388437368 | 0.13910423 | 0.136428445 | -0.007461353 | 0.93664126 |
| THYM | B Cell | 0.558656863 | 1.05E-10 | -0.150117092 | 0.110892219 | 0.556177135 | 1.32E-10 |
| THYM | CD8+ T Cell | 0.364116411 | 6.82E-05 | -0.16213545 | 0.084802154 | 0.314044416 | 0.000667399 |
| THYM | CD4+ T Cell | 0.469056198 | 2.07E-07 | -0.473725751 | 1.51E-07 | 0.112799102 | 0.238507457 |
| THYM | Macrophage | 0.441475264 | 8.80E-07 | -0.099694083 | 0.291268731 | 0.351842331 | 0.000123667 |
| THYM | Neutrophil | -0.218093582 | 0.019749443 | 0.346586969 | 0.000158388 | -0.093295742 | 0.323493927 |
| THYM | Dendritic Cell | 0.585803308 | 7.57E-12 | -0.364571749 | 6.67E-05 | 0.267647084 | 0.003990489 |
| UCEC | Purity | -0.026170038 | 0.654956183 | -0.006176104 | 0.916020326 | 0.025634423 | 0.661576016 |
| UCEC | B Cell | -0.162858212 | 0.005517765 | -0.107319229 | 0.06848764 | -0.125512074 | 0.032932978 |
| UCEC | CD8+ T Cell | 0.209138052 | 0.000344116 | 0.163094407 | 0.005448939 | -0.023876322 | 0.686068598 |
| UCEC | CD4+ T Cell | -0.260550047 | 6.68E-06 | -0.115346896 | 0.049325448 | -0.13282691 | 0.023441879 |
| UCEC | Macrophage | -0.028318569 | 0.629856406 | 0.048451136 | 0.409447271 | -0.104354927 | 0.075005101 |
| UCEC | Neutrophil | 0.22168698 | 0.000130155 | 0.193322605 | 0.000879631 | 0.259870833 | 6.58E-06 |
| UCEC | Dendritic Cell | 0.08071556 | 0.168946296 | 0.038887781 | 0.508025803 | -0.008747209 | 0.88168455 |
| UCS | Purity | -0.334677297 | 0.013375581 | 0.175589082 | 0.204080004 | 0.015775134 | 0.909858038 |
| UCS | B Cell | 0.168775759 | 0.227010097 | -0.082414816 | 0.557417636 | 0.122467708 | 0.382328791 |
| UCS | CD8+ T Cell | 0.230256442 | 0.097177989 | -0.02091762 | 0.881815967 | -0.027463685 | 0.845230262 |
| UCS | CD4+ T Cell | -0.205481826 | 0.139927333 | -0.029412366 | 0.834397847 | -0.05771278 | 0.681454524 |
| UCS | Macrophage | 0.016833068 | 0.904773999 | 0.116666862 | 0.405442209 | 0.113205601 | 0.419610885 |
| UCS | Neutrophil | 0.08887554 | 0.526831925 | -0.19313054 | 0.165878499 | 0.038300706 | 0.785403361 |
| UCS | Dendritic Cell | 0.116641101 | 0.405546624 | -0.035932439 | 0.798385724 | 0.050966023 | 0.717033387 |
| UVM | Purity | 0.221503633 | 0.051299421 | -0.287642069 | 0.010662055 | 0.221997298 | 0.050769633 |
| UVM | B Cell | 0.041114331 | 0.72616735 | 0.301700012 | 0.008525563 | -0.073573271 | 0.530454403 |
| UVM | CD8+ T Cell | 0.395149139 | 0.000375666 | -0.14746919 | 0.200586736 | 0.329674092 | 0.003412745 |
| UVM | CD4+ T Cell | -0.355241359 | 0.00163838 | -0.077433949 | 0.506136831 | -0.126354333 | 0.27675328 |
| UVM | Macrophage | -0.200947922 | 0.111342648 | -0.125341952 | 0.323705705 | 0.007262901 | 0.954578039 |
| UVM | Neutrophil | -0.094842358 | 0.411943845 | 0.663539407 | 4.82E-11 | -0.105584767 | 0.360766041 |
| UVM | Dendritic Cell | 0.184379927 | 0.115809359 | -0.183476104 | 0.117637732 | 0.187030544 | 0.110573333 |
